# Appendicitis

**Published:** 1896-01

**Authors:** 


APPENDICITIS. P. Blakiston, Son & Co., of Philadelphia, announce a book on "Appendicitis." By John B. Deaver, M. D., Assistant Professor of Applied Anatomy, University of Pennsylvania; Assistant Surgeon to the German Hospital, etc. The book will be arranged in a practical and systematic manner. The history, etiology, symptoms, diagnosis, operative treatment, prognosis, and complications of this disease will be given in the order named. It will contain about forty illustrations of methods of procedure in operating, and typical pathological conditions of the appendix, the latter being printed in colors.

